# Plant microbiome-dependent immune enhancing action of *Echinacea purpurea* is enhanced by soil organic matter content

**DOI:** 10.1038/s41598-018-36907-x

**Published:** 2019-01-15

**Authors:** Mona H. Haron, Heather L. Tyler, Suman Chandra, Rita M. Moraes, Colin R. Jackson, Nirmal D. Pugh, David S. Pasco

**Affiliations:** 1 National Center for Natural Products Research, Research Institute of Pharmaceutical Sciences, School of Pharmacy, The University of Mississippi, P.O. Box 1848, University, MS USA; 2 Department of Biology, The University of Mississippi, University, MS USA; 3 Present Address: Crop Production Systems Research Unit, USDA Agricultural Research Service, P.O. Box 350, Stoneville, MS USA; 4 Department of BioMolecular Sciences, Research Institute of Pharmaceutical Sciences, School of Pharmacy, The University of Mississippi, P.O. Box 1848, University, MS USA

## Abstract

We previously demonstrated that extracts from *Echinacea purpurea* material varied substantially in their ability to activate macrophages *in vitro* and that this variation was due to differences in their content of bacterial components. The purpose of the current study was to identify soil conditions (organic matter, nitrogen, and moisture content) that alter the macrophage activation potential of *E. purpurea* and determine whether these changes in activity correspond to shifts in the plant-associated microbiome. Increased levels of soil organic matter significantly enhanced macrophage activation exhibited by the root extracts of *E. purpurea* (p < 0.0001). A change in soil organic matter content from 5.6% to 67.4% led to a 4.2-fold increase in the macrophage activation potential of extracts from *E. purpurea*. Bacterial communities also differed significantly between root materials cultivated in soils with different levels of organic matter (p < 0.001). These results indicate that the level of soil organic matter is an agricultural factor that can alter the bacterial microbiome, and thereby the activity, of *E. purpurea* roots. Since ingestion of bacterial preparation (e.g., probiotics) is reported to impact human health, it is likely that the medicinal value of *Echinacea* is influenced by cultivation conditions that alter its associated bacterial community.

## Introduction

Evidence from our lab^1–4^ and others^5,6^ supports the theory that the efficacy of *E. purpurea* against respiratory infections is dependent, at least in part, on its bacterial community (microbiome). Bacterial components of this microbiome can directly impact immune function^1–5^ and plant-endophyte interactions can alter secondary metabolite production of the anti-inflammatory alkylamides^6^. The immune-activating bacterial components may exhibit therapeutic effects against respiratory infection comparable to those reported in clinical research on probiotic bacteria^7^. In addition, the anti-inflammatory alkylamides may provide symptomatic relief to colds and the flu, analogous to NSAIDs.

In our previous studies we reported that the bacterial components lipopolysaccharide (LPS) and Braun-type lipoproteins within extracts of *E. purpurea* and other botanicals were responsible for essentially all of the *in-vitro* activation of monocytes/macrophages^1^. Consistent with these findings, our later studies showed that the level of *in vitro* macrophage activation exhibited by an *E. purpurea* extract could be predicted by calculating the sum of activities contributed by the prevalence and types of Proteobacteria within the plant material^4^. Furthermore, we found that root and aerial extracts from *E. purpurea* and *E. angustifolia*, obtained from six distinctly different regions in North America, exhibited substantial variation in macrophage stimulatory activity (up to 200-fold). The majority of detected activity was due to changes in levels of LPS and bacterial Braun-type lipoproteins, and differences in post-harvesting conditions did not appear to be responsible for the observed variation in activity^2^. Follow-up research indicated that the *Echinacea* sourced from the six geographical locations also exhibited variation in both total bacterial load (up to 52-fold) and composition of the bacterial community^3,4^.

A growing body of literature suggests that environmental and agronomic conditions shape the microbiome of plants. Bacteria in soil are a reservoir for plant endophytes in that communities of bacteria within the rhizosphere are able to colonize roots and other plant tissues. Soil containing high levels of organic matter and moisture support high soil microbial load^8,9^ and increased microbial biomass can lead to enhanced microbial colonization of roots^10^. An opposite effect has been observed with nitrogen fertilization - high rates of application reduce colonization of plants by bacterial endophytes^11,12^. In light of these findings, the objective of the present study was to determine the contribution of soil organic matter, nitrogen fertilization, and moisture content on the immune enhancing activity of *E. purpurea* and evaluate shifts in the plant-associated bacterial community that could be responsible for the activity changes.

## Results

**Figure 1:**
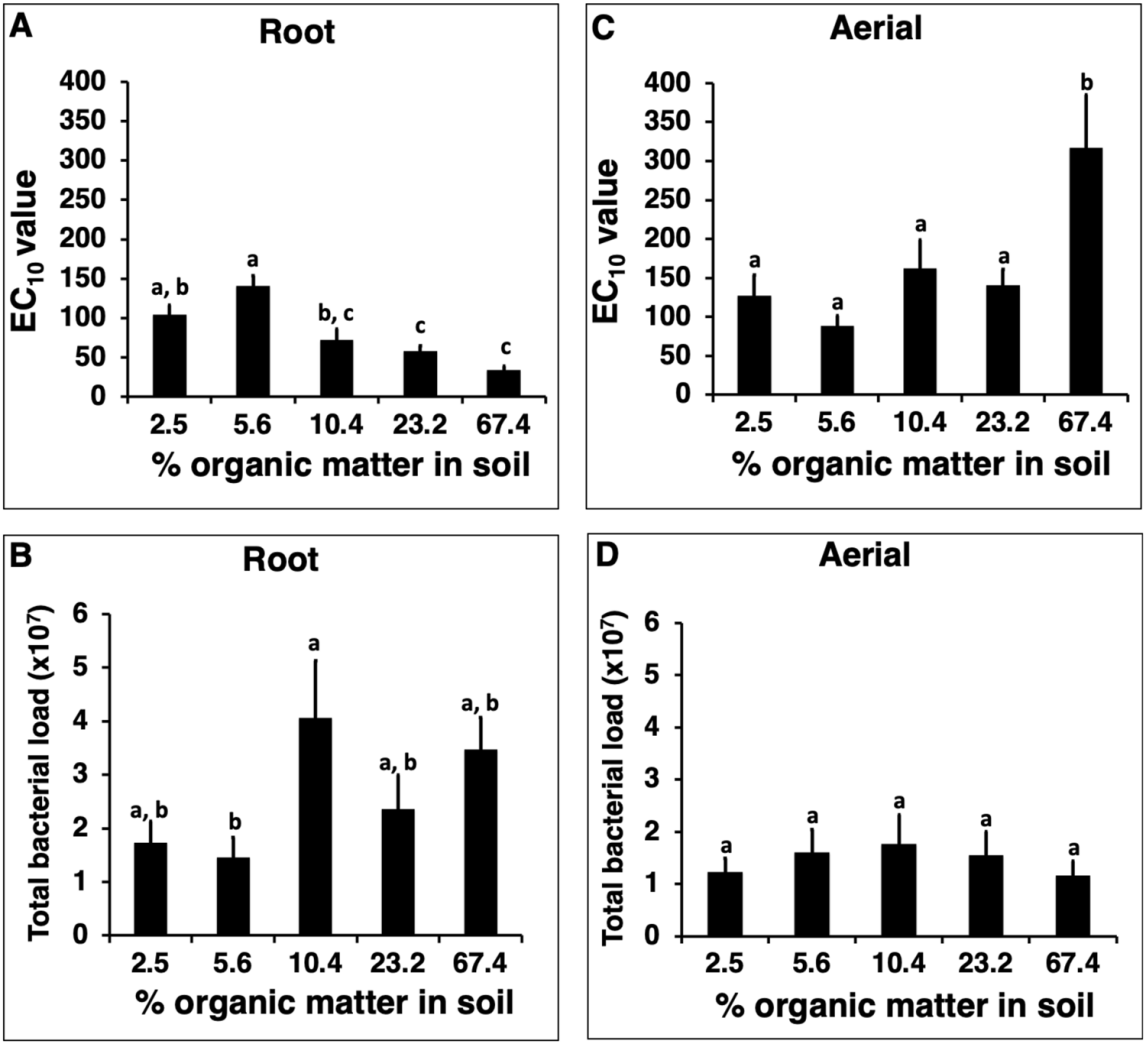
Effect of cultivating *Echinacea purpurea* in soils containing different levels of organic matter on *in vitro* macrophage stimulatory activity and bacterial load of the plant material. Soil with five different levels of organic matter (determined gravimetrically after combustion) was used. *E. purpurea* seedlings were grown in pots for five months, with six pots with three plants per pot, for each soil type. Dried roots (**A**) and aerial (**C**) material were extracted with 4% SDS and extracts evaluated for activity (macrophage TNF-alpha production, expressed as EC_10_ values). Total bacterial load (**B,D**) represents cells per gram of dried plant material. Bars represent mean values ± standard error. Treatments with different letters are significantly different (*p* < 0.05).

For Experiment 1, cultivating *E. purpurea* in soil containing the higher levels of organic matter (10.4–67.4%) compared to lower levels (2.5% and 5.6%) resulted in enhanced *in vitro* macrophage activation (i.e., lower EC_10_ values) by extracts of the root material (Fig. 1A). Mean activity of extracts from plants cultivated in soil containing 2.5% organic matter based on dry weight was significantly lower than plants grown in 23.2% and 67.4% organic matter (*p* = 0.026 and *p* = 0.0002, respectively). Similarly, mean activity from plants cultivated in 5.6% was significantly lower than plants grown in 10.4%, 23.2% and 67.4% organic matter (*p* = 0.0002, *p* < 0.0001 and *p* < 0.0001, respectively). The largest difference in mean *in vitro* macrophage activation (4.2-fold) was observed between plants grown is soil that differed by 12 times in the content of organic material (67.4% versus 5.6%).

Root tissue from plants cultivated in soil containing a higher organic content (10.4–67.4%) had a higher bacterial load than roots grown in lower organic matter soil (2.5% and 5.6%), with higher organic matter treatments yielding bacterial loads of 2.4–4.1 × 10^7^ cells per gram of root compared to 1.5–1.7 × 10^7^ bacterial cells per gram in roots grown under low organic matter (Fig. 1B). The data for both activity and bacterial load suggests a possible threshold where plants cultivated in soil containing organic matter at or above 10% are significantly different in these parameters. Comparison of roots grown in lower organic matter soil (2.5% and 5.6% treatments) to those cultivated in higher organic matter treatments (10.4%, 23.2%, and 67.4%), showed that the higher organic matter soils resulted in significantly higher bacterial loads (*p* = 0.005). However, for individual treatments the only statistically significant difference between conditions was observed between roots cultivated in soil containing 5.6% organic matter versus 10.4% organic matter (*p* = 0.04).

For aerial *E. purpurea* tissue, the only significant effects of soil organic matter treatments on activity (Fig. 1C) or bacterial load (Fig. 1D) was that lower activity was found in plants cultivated in soil containing 67.4% organic matter versus plants grown in soils with lower levels of organic matter (*p* < 0.03). Aerial portions of plants under this organic matter treatment also had the lowest mean bacterial loads, but this, as with the other effects of organic matter on aerial components of the plant, was not significant.

**Figure 2:**
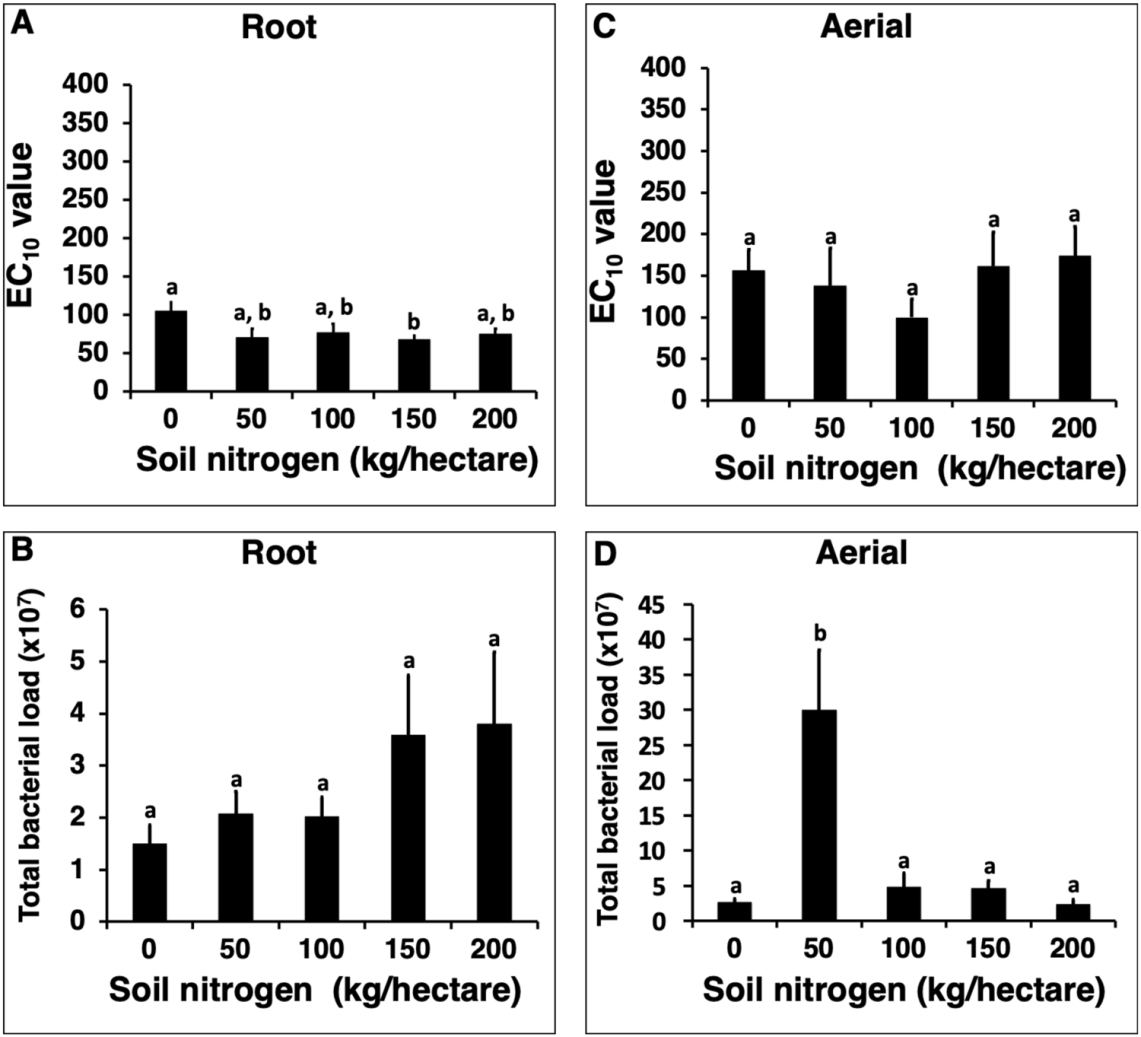
Effect of cultivating *Echinacea purpurea* in soils containing different levels of nitrogen fertilization on *in vitro* macrophage stimulatory activity and bacterial load of the plant material. Soil with five levels of nitrogen fertilization (equivalent to 0, 50, 100, 150, and 200 kg/hectare) was used. *E. purpurea* seedlings were grown in pots for five months, with six pots with three plants per pot, for each soil type. Dried roots (**A**) and aerial (**C**) material were extracted with 4% SDS and extracts evaluated for activity (macrophage TNF-alpha production, expressed as EC_10_ values). Total bacterial load (**B,D**) represents cells per gram of dried plant material. Bars represent mean values ± standard error. Treatments with different letters are significantly different (*p* < 0.025).

**Figure 3:**
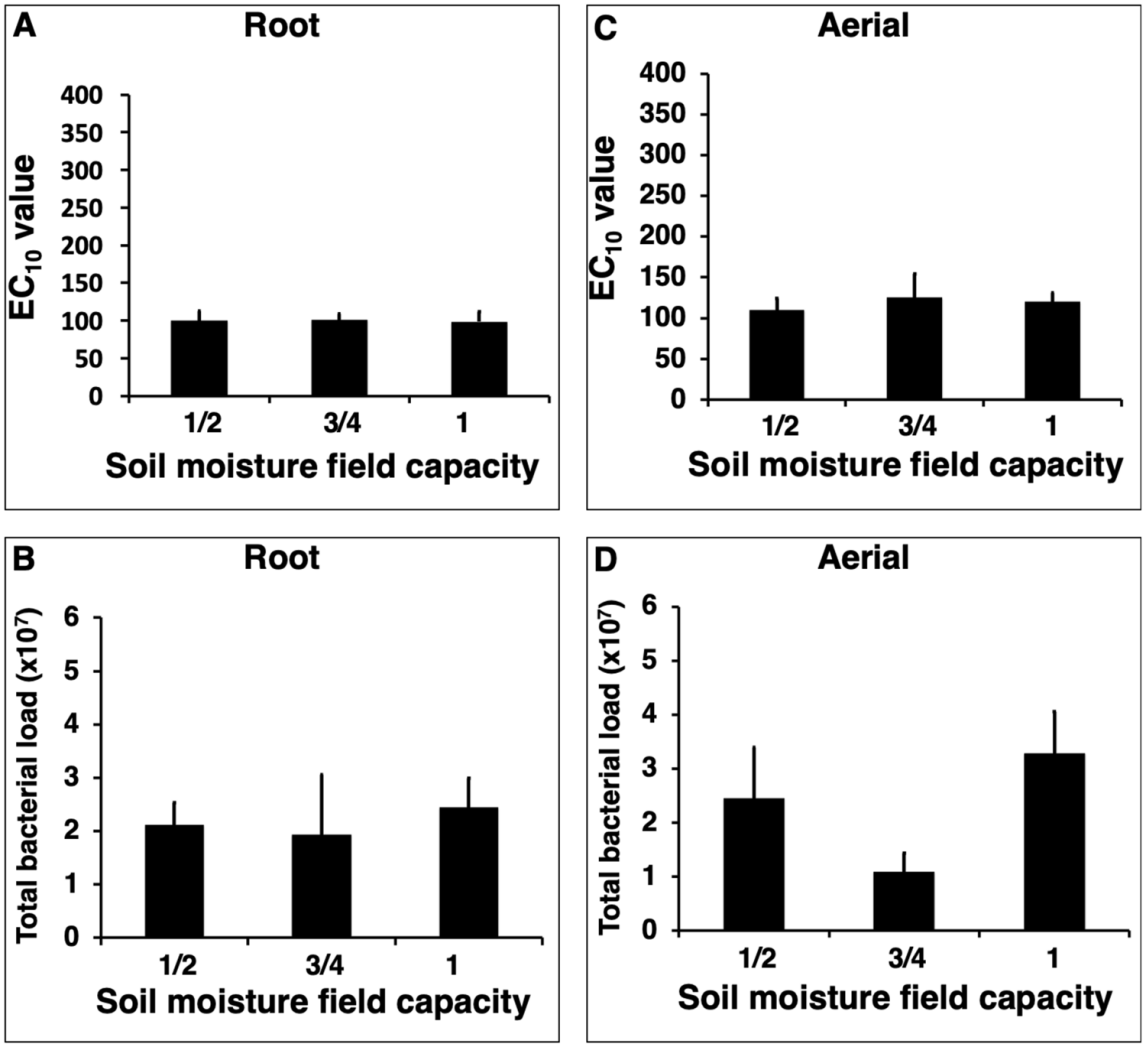
Cultivating *Echinacea purpurea* in soils containing different levels of moisture has no effect on *in vitro* macrophage stimulatory activity and bacterial load of the plant material. Seedlings were grown for five months in soil containing three different levels of moisture (six pots per treatment, with three plants per pot). All roots (**A**) and aerial (**C**) material were extracted with 4% SDS and extracts evaluated for activity (macrophage TNF-alpha production, expressed as EC_10_ values). Total bacterial load (**B,D**) represents cells per gram of dried plant material. Bars represent mean values ± standard error.

Cultivating *E. purpurea* in soil containing different levels of nitrogen (Experiment 2) or moisture (Experiment 3) did not influence plant extract activity or total bacterial load (Figs 2 and 3). The only statistically significant differences were observed in the nitrogen treatment experiment where root extracts from plants cultivated in soil supplemented with 150 kg/hectare of nitrogen exhibited higher mean activity than roots grown with no additional nitrogen supplementation (*p* = 0.02, Fig. 2A). Mean bacterial load was dramatically increased in aerial tissue from plant cultivated in soil supplemented with 50 kg/hectare of nitrogen versus plants grown in soils with lower and higher levels of nitrogen (*p* < 0.0001, Fig. 2D).

**Figure 4:**
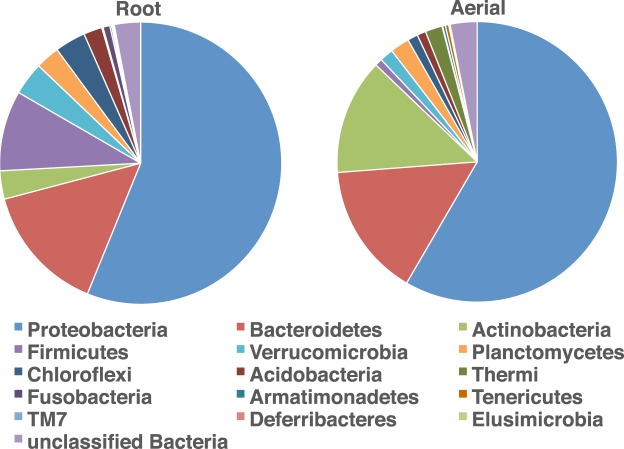
Average bacterial microbiome composition in *E. purpurea* root and aerial material. Proportions of taxa (phyla) shown are derived from a total of 13,231,553 partial 16S rRNA gene sequences obtained from the plants that were cultivated using the different agronomic conditions detailed in Figs 1–3. A total of 78 plants were analyzed (one plant from each pot: n = 30 for Experiment 1, n = 30 for Experiment 2, and n = 18 for Experiment 3).

For analysis of the *E. purpurea* microbiome, one plant per pot was analyzed for the treatment conditions in each experiment (30 plants for Experiment 1, 30 plants for Experiment 2 and 18 plants for Experiment 3). After initial processing, alignment, and removal of sequences identified as chimeras or plant chloroplasts a total of 1,352,517 final valid bacterial 16S rRNA gene sequences were recovered from root and aerial samples of these 78 *E. purpurea* plants. Sequences were then binned into 8,805 distinct operational taxonomic units (OTUs) based on 97% sequence similarity, spanning 16 different bacterial phyla. In both the root and aerial *E. purpurea* tissues, the majority of bacteria identified were consistently members of the Proteobacteria and Bacteroidetes (Fig. 4). On average, Proteobacteria comprised 56% (roots) and 58% (aerial), while Bacteroidetes made up 15% (both roots and aerial) of the sequences recovered.

**Figure 5:**
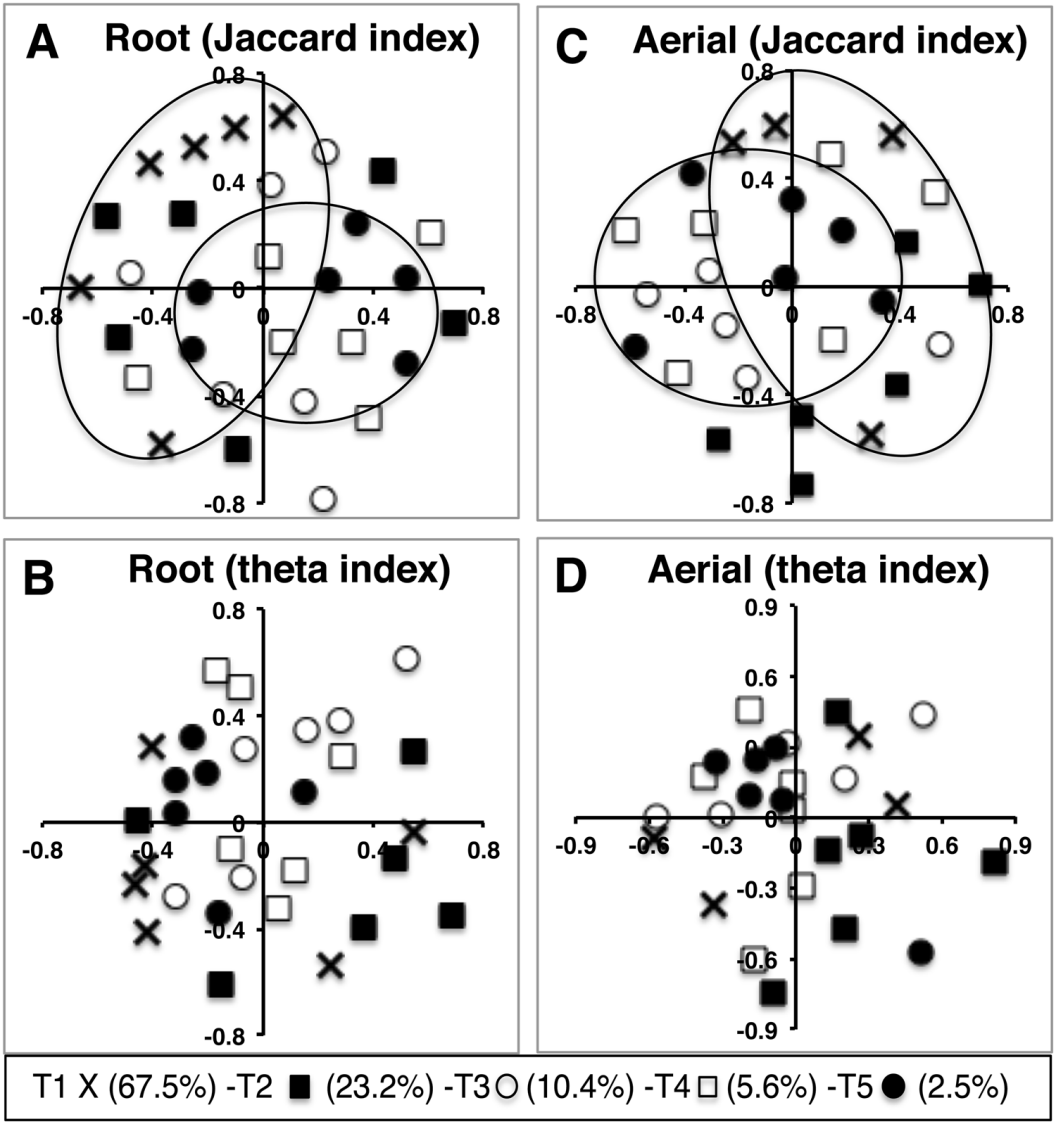
NMDS ordinations showing the influence of cultivating *E. purpurea*, in soils containing different levels of organic matter, on bacterial community structure of the plant material. Ordinations show communities derived from root material compared using the Jaccard index (**A**, stress = 0.38) and theta index (**B**, stress = 0.24), and aerial material compared by the Jaccard index (**C**, stress = 0.37) and theta index (**D**, stress = 0.18). Eclipses in panel **A** and **C** show sets of samples that were significantly different from each other (*p* < 0.005). “T” represents treatment condition and percentages indicate level of soil organic matter.

Different levels of soil nitrogen fertilization or soil moisture did not result in significant changes in bacterial community structure within the plant material (Table 1) so we did not pursue these investigations further. However, changes in the level of soil organic matter resulted in differences in the bacterial community structure of both root and aerial *E. purpurea* (Table 1), a pattern that was also apparent in non-metric multidimensional scaling (NMDS) ordinations of microbiome composition under different organic matter treatments (Fig. 5). NMDS ordinations based on the Jaccard dissimilarity index (presence-absence of OTUs), showed some grouping of bacterial communities under the highest and lowest treatments of % organic matter, in both root and aerial samples of *E. purpurea* (Fig. 5), although these were not as prevalent for ordinations based on the theta index. Analysis of molecular variance (AMOVA) and analysis of similarity (ANOSIM) strongly supported significant differences in community composition among % organic matter treatments for both types of samples (p < 0.001). In terms of pairwise comparisons between organic matter treatments, there was a significant difference between bacterial communities at 2.5% and 67.4% organic matter for the aerial samples (AMOVA: *p* = 0.002) and a strong suggestion of the same differences in root samples (AMOVA: *p* = 0.006) (Table 1 and Supplementary Table S1).

**Table 1:** Community similarity (as assessed based on presence-absence of bacterial OTUs, Jaccard, or relative abundance of OTUs, theta, metrics and analyzed by AMOVA) for bacterial communities in *E. purpurea* under the different cultivation conditions tested.

Plant part	Soil condition	p value		Jaccard Pairwise p value			
		Jaccard	Theta	T1 vs T5	T1 vs T4	T2 vs T5	T2 vs T3
Root	Organic matter	<0.001*	0.044*	0.006	0.008	0.011	—
	Nitrogen	0.208	0.366	—	—	—	—
	Moisture content	0.436	0.373	—	—	—	—
Aerial	Organic matter	<0.001*	0.21	0.002**	0.009	0.007	—
	Nitrogen	0.119	0.783	—	—	—	—
	Moisture content	0.05	0.486	—	—	—	0.014

% organic matter in soil T1 (67.5%) – T2 (23.2%) – T3 (10.4%) – T4 (5.6%) – T5 (2.5%), soil nitrogen fertilization T1 (200 Kg/h) – T2 (150 Kg/h) – T3 (100 Kg/h) – T4 (50 Kg/h) – T5 (0 Kg/h) and soil moisture field capacity T1 (full field) – T2 (3/4 of field) – T3 (1/2 field). *Statistically significant difference among treatments in each condition using an alpha of 0.05.

^**^Statistically significant pairwise comparison using an alpha of 0.005.

Indicator analysis identified the specific OTUs that differed significantly in their relative abundance across organic matter treatments for both root and aerial samples. For roots, 30 OTUs, belonging to seven bacterial phyla, showed significant differences in their relative abundance between different levels of soil organic matter (Table 2). Of those, 23 OTUs were proportionally more abundant in the elevated (67.4%) organic matter samples. Aerial samples showed significant variation in the relative abundance of 21 OTUs, belonging to seven bacterial phyla, across organic matter treatments, with Actinobacteria and Proteobacteria being common in all but the two lowest (2.5%, 5.6%) organic matter concentrations (Table 3).

**Table 2:** Classification, distribution, and relative abundance of 16S rRNA-defined bacterial OTUs recovered from root samples of *E. purpurea* treated with five different concentrations of organic matter in soil.

	OTU	Phyla	Family or genus	Average percent/group				
				T1	T2	T3	T4	T5
T1	0211	Acidobacteria	Group 4	0.151*	0.000	0.002	0.000	0.000
	0777	Acidobacteria	Group 6	0.100*	0.027	0.008	0.000	0.000
	0140	Actinobacteria	Streptomyces	0.945*	0.096	0.141	0.198	0.053
	0470	Actinobacteria	Conexibacter	0.224*	0.023	0.049	0.022	0.000
	0077	Actinobacteria	Aeromicrobium	0.193*	0.117	0.006	0.000	0.024
	0307	Bacteroidetes	Chryseolinea	0.165*	0.109	0.035	0.019	0.013
	0299	Bacteroidetes	Chryseolinea	0.126*	0.059	0.017	0.005	0.040
	0124	Bacteroidetes	Chitinophagaceae	0.308*	0.043	0.018	0.014	0.144
	0094	Bacteroidetes	Flavobacterium	0.668*	0.039	0.165	0.128	0.122
	0500	Bacteroidetes	Filimonas	0.120*	0.000	0.000	0.010	0.008
	0332	Chloroflexi	Ktedonobacter	0.112*	0.084	0.032	0.006	0.011
	0342	Chloroflexi	Heliothrix	0.131*	0.061	0.041	0.044	0.019
	0801	Chloroflexi	Levilinea	0.073*	0.017	0.010	0.007	0.012
	0362	Chloroflexi	Bellilinea	0.115*	0.020	0.027	0.057	0.000
	0478	Firmicutes	Unclassified	0.103*	0.069	0.028	0.000	0.011
	0649	Firmicutes	Aneurinibacillus	0.082*	0.000	0.000	0.006	0.000
	0759	Planctomycetes	Zavarzinella	0.054*	0.000	0.006	0.000	0.028
	0892	Planctomycetes	Thermogutta	0.072*	0.028	0.013	0.005	0.000
	0164	Alphaproteobacteria	Sphingopyxis	0.782*	0.019	0.194	0.021	0.024
	0232	Alphaproteobacteria	Sphingobium	0.178*	0.002	0.028	0.079	0.006
	0904	Alphaproteobacteria	Oceanibaculum	0.088*	0.016	0.006	0.007	0.000
	1040	Verrucomicrobia	Terrimicrobium	0.055*	0.000	0.000	0.000	0.000
	0400	Verrucomicrobia	Spartobacteria	0.123*	0.011	0.003	0.036	0.005
T3	0506	Firmicutes	Bacillus	0.000	0.015	0.091*	0.006	0.006
	0005	Gammaproteobacteria	Enterobacteriaceae	2.679	8.240	10.270*	6.820	3.468
	0184	Gammaproteobacteria	Stenotrophomonas	0.029	0.000	0.080*	0.056	0.025
T5	0259	Bacteroidetes	Flavisolibacter	0.034	0.026	0.004	0.050	0.122*
	0198	Alphaproteobacteria	Asticcacaulis	0.139	0.057	0.016	0.095	0.296*
	0246	Betaproteobacteria	Cupriavidus	0.053	0.058	0.085	0.011	0.469*
	0644	Verrucomicrobia	Subdivision 3	0.017	0.000	0.000	0.000	0.139*

OTUs shown were detected in at least two out of six replicates per treatment condition. Percent organic matter in soil: T1 (67.5%) – T2 (23.2%) – T3 (10.4%) – T4 (5.6%) – T5 (2.5%). A total of 48,197 16 S rRNA gene sequences were analyzed for treatment T1, 26,471 for T2, 64,655 for T3, 18,555 for T4, and 40,933 for T5.

Significance between treatments indicated by *p < 0.05.

**Table 3:** Classification, distribution, and relative abundance of 16S rRNA-defined bacterial OTUs recovered from aerial samples treated with 5 different concentrations of organic matter in soil. OTUs shown were detected in at least 2 out of 6 replicates per treatment condition.

	OTU	Phyla	Family or genus	Average percent/group				
				T1	T2	T3	T4	T5
T1	0226	Actinobacteria	Actinoplanes	0.564*	0.037	0.000	0.000	0.000
	1757	Bacteroidetes	Spongiimonas	0.102*	0.000	0.000	0.000	0.000
	0423	Alphaproteobacteria	Lentibacter	0.239*	0.051	0.059	0.039	0.094
T2	0140	Actinobacteria	Streptomyces	0.061	0.161*	0.013	0.000	0.000
	0128	Bacteroidetes	Niastella	0.086	0.189*	0.014	0.000	0.000
	0158	Chloroflexi	Anaerolineaceae	0.017	0.150*	0.014	0.012	0.000
	0110	Chloroflexi	Kallotenue	0.008	2.638*	0.114	0.082	0.014
	0269	Chloroflexi	Ktedonobacter	0.000	0.110*	0.004	0.000	0.000
	0025	Betaproteobacteria	Comamonadaceae	0.174	0.506*	0.053	0.270	0.089
	0125	Gammaproteobacteria	Acinetobacter	0.081	0.485*	0.019	0.030	0.077
T3	0415	Actinobacteria	Conexibacter	0.030	0.000	0.093*	0.026	0.009
	0176	Actinobacteria	Nocardioides	0.183	0.069	0.527*	0.198	0.230
	0250	Bacteroidetes	Hymenobacter	0.000	0.035	0.239*	0.165	0.030
	0124	Bacteroidetes	Chitinophagaceae	0.139	0.054	0.258*	0.079	0.072
	0623	Bacteroidetes	Hymenobacter	0.000	0.000	0.117*	0.000	0.015
	0773	Alphaproteobacteria	Ancylobacter	0.000	0.015	0.066*	0.000	0.000
	0156	Betaproteobacteria	Methylophilus	0.025	0.024	0.179*	0.092	0.081
T4	0265	Planctomycetes	Tepidisphaera	0.010	0.000	0.007	0.381*	0.021
	0564	Planctomycetes	Unclassified	0.005	0.001	0.000	0.080*	0.000
T5	1006	Ignavibacteriae	Melioribacter	0.000	0.000	0.000	0.000	0.127*

Percent organic matter in soil: T1 (67.5%) – T2 (23.2%) – T3 (10.4%) – T4 (5.6%) – T5 (2.5%). A total of 12,204 16S rRNA gene sequences were analyzed for treatment T1, 39,556 for T2, 30,319 for T3, 23,518 for T4, and 27,428 for T5.

Significance between treatments indicated by *p < 0.05.

## Discussion

Changes in soil organic matter content, but not levels of nitrogen or water, had a significant influence on the macrophage activation potential of root extracts from *E. purpurea*. There appears to be a threshold effect, where roots grown in soil containing organic matter at or above about 10% dry weight exhibited significantly higher activity and higher total bacterial load. Furthermore, soil organic matter content was the only agronomic variable that significantly altered the microbiome of both root and aerial tissue. We have previously demonstrated that essentially all *in vitro* macrophage activation exhibited by *Echinacea* is due to bacterially-derived components^1^, so it is likely that the observed shifts in the *Echinacea* microbiome are responsible for the enhanced activity of the root extracts.

All plants contain communities of bacteria that are associated with roots and aerial tissues^13^. A rapidly growing area of research is providing new understanding of the composition of plant microbiomes and factors that guide their composition. Evidence indicates that the root microbiome is predominantly assembled from the bacteria present within the soil in which the plant is grown^14^. We derived a similar conclusion based on our initial research from changes in activity of *Echinacea* plants grown in tissue culture verses soil^15^. Clones were identified that contained different, but stable, bacterial populations as evidenced by high and low levels of macrophage-activating bacterial components detected in their extracts. However, the high and low activity clones exhibited the same activity after several months of cultivation in typical potting soil, a result that indicated that soil is a major factor in determining the composition of the *Echinacea purpurea* microbiome.

The root microbiome is usually less diverse than the rhizosphere, and bacterial colonization of plants is selective with certain bacterial taxa more likely to colonize and be retained in plant tissue^16,17^. For example, compared to the surrounding soil, the root microbiome is generally enriched for members of the Proteobacteria, whereas plant-associated members of the Acidobacteria and Gemmatimonadetes are less common than in soil^18,19^. Results from the current study show that soil chemical composition is important to the plant microbiome selection process, with changes in soil organic matter content resulting in differences in the plant-associated bacterial community. Plants cultivated in soil containing higher amounts of the same organic matter acquired significantly different bacterial consortia in their root and aerial tissues. This selection for different microbiome composition under different levels of soil organic matter could represent a different pool of potential bacterial inoculants in the soil, or a change in the plant-microbiome selection criteria when plants are grown under different levels of organic matter.

Based on findings from our previous research, it is likely that the shifts in the root bacterial community structure of *Echinacea* cultivated in soil containing higher organic matter are responsible for increased macrophage stimulating activity exhibited by this tissue. We have determined that the *in vitro* activity of *Echinacea* extracts detected in monocytes/macrophages is essentially all due to the bacterial components LPS and Braun-type lipoproteins^1^. Therefore, the increased activity of roots cultivated in higher organic matter would logically be derived from changes in bacterial components derived from shifts in the tissue’s microbiome. We have also reported that there are two factors that determine *Echinacea* extract activity – total bacterial load and activity exhibited by each type of bacteria (which can vary by over 8000-fold). Using these two factors, we found that *Echinacea* extract activity can be accounted for by the activities and prevalence of Proteobacteria members colonizing this plant^4^. In the current study we have suggestive evidence that both bacterial load (Fig. 1B) and the type of bacteria (Fig. 5, Tables 1 and 2) are responsible for the increased root activity observed in the plants cultivated in soil containing higher organic matter. Plants cultivated in higher levels of organic matter had roots that contained a higher percentage of various taxa (e.g., *Aeromicrobium*, *Flavobacterium*, *Sphingopyxis*, *Sphingobium* from treatment 1, and *Stenotrophomonas* from treatment 3; Table 2) that we have previously determined to be robust stimulators of *in vitro* macrophage activation^4^. However, we do not know the activity exhibited by the other taxa listed in Table 2 since we were unable to isolate those bacteria (or closely related taxa) from *Echinacea* tissue for testing during our earlier culturing attempts^4^. Therefore, we do not know the extent of activity that is contributed by each bacterial member to the overall increase in macrophage stimulation observed from extracts of roots cultivated in soil containing different levels of organic matter.

Research is beginning to evaluate whether plants from different geographical locations vary in their microbiome^18^. Although similarities are found at the phylum level, large variation has been observed at finer taxonomic levels^20^. In agreement with these findings, we have found that bulk *Echinacea* material sourced from six different geographical locations in North America varied in total bacterial load by 52-fold^3^ and exhibited variation in community members at the genus level^4^. Extracts from the root and aerial tissues from these six different locations exhibited variation in macrophage-stimulating activity (up to 200-fold), and essentially all activity was due to changes in the levels of bacterial components derived from variations in the plant microbiome^2^. In the current study, increasing the level of soil organic matter by 12 times resulted in a 4.2-fold increase in macrophage stimulatory activity of extracts from *Echinacea* root tissue. Although these data indicate that level of soil organic matter is an important variable, clearly other agronomic/environmental factors are also responsible for determining the 200-fold variation we have observed in *Echinacea* cultivated in different geographical locations. Additional factors may include type of organic matter in the soil, cultivation practices and environmental variables, and post-harvesting procedures.

Plant microbiome research has focused on the development of strategies for improving agricultural production, plant health, and ecosystem dynamics. An area that has been generally overlooked is the human health implications of consuming the microbial communities associated with plants in our diet. Plants can be colonized by up to 10^4^ and 10^10^ microorganisms per gram of tissue and, when consumed, these microorganisms could alter the composition of our gut microbiome as well as influence our immune system^21^. In our research, we have found that a normal dose of *Echinacea* contains between 6.4 × 10^6^ to 3.3 × 10^8^ bacterial cells per g of dry plant material and is comparable to a therapeutic dose of probiotic bacteria^3^. The type of bacteria comprising plant microbiomes is probably another critical factor in determining the positive or negative implications of ingesting plants. Probiotic research has clearly illustrated that even strain differences can have a dramatic influence on therapeutic efficacy. For example, protection against influenza viral infection in mice was observed after oral administration of a *Lactobacillus plantarum* strain inducing high cytokine production *in vitro*, whereas no protection was observed for strains exhibiting inhibition of or low cytokine production^22^. It is therefore possible that the efficacy of *Echinacea* is dependent, at least in part, on the structure of its associated bacterial community. Future research is needed to fully understand the effect of domestication and modern agricultural practices on the microbiome of *Echinacea*, as well as other crops, and the implications of these effects on human health.

## Methods

### Cultivation, treatment and harvesting conditions of *E. purpurea* plants

*Echinacea purpurea* (L.) Moench (Asteraceae) seeds (accession PI 631307) were provided by The North Central Regional Plant Introduction Station at Iowa State University (Ames, IA). Seeds were sown in plastic trays in May 2013 at the University of Mississippi medicinal plant garden. Most of the seeds germinated in 2–3 weeks. After gaining a height of ~10–12 cm, plants were transplanted to bigger pots (28 cm tall, 29 cm diameter at top and 24 cm diameter at bottom). For each treatment condition, six pots were filled with appropriate soil type with three seedlings/pot. Pots were arranged in a complete randomized block design and plants were grown in the full sun for Experiments 1 and 2, and in the green house for Experiment 3 (to control soil moisture level). All plants were harvested after five months of treatment. Aerial parts were separated from roots and roots were then washed extensively to remove soil. Aerial parts consisted of stems and leaves only (flowers were not included). Plant parts were immediately frozen at −80 °C to prevent postharvest growth or contamination with bacteria. Frozen plant parts were freeze-dried, ground to powder and stored at −20 °C.

#### Experiment 1. Testing the contribution of organic matter content of the soil

Formulations of soil, with increasing levels of organic matter, were created by mixing washed sand with Pro-mix Ultimate Organic Mix (Hummert International Co., Earth City, MO) at various ratios: 20, 40, 60, 80 and 100% Pro-mix by volume. Level of soil organic matter content was determined gravimetrically by drying (75 °C, 48 h), followed by combustion (500 °C, 2 h). All the plants were irrigated manually and equally on a daily basis.

#### Experiment 2. Testing the contribution of nitrogen fertilization

Seedlings were planted in soil low in nitrogen that consisted of one volume Pro-Mix BRK (Hummert International Co.) and three volumes washed sand (to decrease nitrogen content). Five levels of ammonium nitrate were added to pots at three time points, once at seedling transplantation and the next two at monthly intervals. Levels of nitrogen added were equivalent to 0, 50, 100, 150, and 200 kg/hectare. All the plants were irrigated manually and equally on a daily basis.

#### Experiment 3. Testing the contribution of soil moisture

Plants were grown in Pro-Mix BRK soil. Levels of soil moisture tested were: full field capacity, ¾ of field capacity and ½ of field capacity. Water was supplied on a regular basis depending on the evaporation rate.

### Determination of macrophage activation by *E. purpurea* plant extracts

Crude biochemical extracts were prepared for all the *E. purpurea* plant samples as previously described^3^. In brief, plant material was extracted with 95% ethanol to remove anti-inflammatory compounds. Dried ethanol-extracted plant material was then further extracted with 98 °C water containing 4% SDS. SDS was removed using SDS-out reagent in the presence of 1% octylglucoside and crude extracts evaluated for activity. Macrophage activation was assessed by measuring tumor necrosis factor α (TNF-α) levels in culture supernatants from RAW 264.7 cells (ATCC) incubated with crude extracts for 18–24 h. The level of TNF-α was determined using enzyme-linked immunosorbent assays (ELISA) (R&D Systems) following the manufacturer’s protocol. Macrophage activation for plant material is reported as an EC_10_ value that represents the concentration (μg/mL) of plant material required to induce TNF-α production to 10% of levels achieved by ultrapure *E. coli* LPS 0111:B4 strain (InvivoGen, 1 mg/mL corresponds to 1 × 10^6^ EU/mL) tested at 100 ng/mL.

### *E. purpurea* total bacterial load estimation

Total bacterial load in all *E. purpurea* tissue samples was determined through a PCR-based method as described previously^3^. DNA was extracted from 50 mg of ground, lyophilized *E. purpurea* tissue samples using PowerPlant DNA isolation kits (MoBio). Prior to extraction, samples were hydrated with 150 μL sterile water. DNA extracts were cleaned using PowerClean DNA Cleanup Kits (MoBio) to remove potential PCR inhibitors. A portion of the bacterial 16S rRNA gene was amplified using primers 799f (5′-AACMGGATTAGATACCCKG-3′) and 1492r (5′-GGTTACCTTGTTACGACTT-3′) that exclude the coamplification of chloroplast DNA and yield a 735 bp bacterial product and a 1090 bp mitochondrial product when used to amplify DNA extracted from plant material^23^. DNA amplifications were conducted as previously described^3^. Bacterial loads were determined by comparing the intensity of the 735 bp bacterial band from *E. purpurea* extracts to a standard curve of DNA extracted and amplified from known quantities of bacteria as described previously^3^.

### Determination of *E. purpurea* bacterial community structure

Analysis of the bacterial community associated with *E. purpurea* was conducted on the same DNA extracts used for bacterial load determination. One plant per pot was analyzed for the treatment conditions in each experiment (a total of 78 root and 78 aerial samples). A dual-index barcoding approach was used for Illumina next generation sequencing where each sample was amplified using primers that target a 250 bp section of the V4 variable region of the bacterial 16S rRNA gene^24^. Procedures followed those outlined by Jackson *et al*.^24^ and amplification conditions described by Kozich *et al*.^25^. Amplification products from all samples were pooled and spiked with 5% PhiX to increase nucleotide base diversity prior to sequencing. The final library was sequenced on an Illumina MiSeq instrument, via two index sequencing reads, at the University of Mississippi Medical Center Molecular and Genomics Core Facility.

Raw sequence files (fastq files) were accessed using the bioinformatics software Mothur^26^, and were processed and analyzed following the procedures recommended by Kozich *et al*.^25^ and Jackson *et al*.^24^. Briefly, contigs were assembled from paired end reads and screened to only include those with a maximum length of 275 bp and no base ambiguities. Sequences were aligned against the SILVA 16S rRNA database (v132)^27^ and misaligned sequences were deleted. Sequences were clustered together by 1% sequence similarity to account for potential amplification and sequencing errors, and chimeras removed using UCHIME^28^. Valid sequences were classified according to those in the RDP 16S rRNA database, version 16, after which any sequences classified as being other than bacterial were removed from the dataset. Remaining sequences were grouped into operational taxonomic units (OTUs) based on >97% sequence similarity.

### Statistical analysis

Analysis of variance (ANOVA) followed by Tukey-Kramer Honestly Significant Difference (HSD) test was conducted to determine the effects of different levels of soil organic matter content, nitrogen fertilization, and soil moisture on macrophage stimulating activity and bacterial load of root and aerial tissue. These analyses were assessed using an alpha of 0.05 and performed in JMP version 11.2.0. (SAS Institute Inc.). Analysis of microbiome patterns was conducted in Mothur. Presence or absence of specific OTUs in each sample were used to compare bacterial communities using the Jaccard index of dissimilarity, while the relative proportion of each OTU in each sample was used to compare communities using the Yue & Clayton theta index. Community comparisons were visualized using NMDS ordinations while specific comparisons of community structure were conducted using AMOVA and ANOSIM. Differences in the relative abundance of individual bacterial phyla or OTUs between treatments were analyzed by Indicator analysis function in Mothur.

## Supplementary information

Supplementary Table S1

## Data Availability

Raw data is available upon request.
