# Supplementary material for: Plant microbiome-dependent immune enhancing action of Echinacea purpurea is enhanced by soil organic matter content
Source: Sci Rep. 2019 Jan 15;9:136. doi: 10.1038/s41598-018-36907-x (PMC6333828; doi:10.1038/s41598-018-36907-x)
Supplement: Supplementary file 1 — Supplementary Table S1 [file 41598_2018_36907_MOESM1_ESM.pdf]

Supplementary Information

Title of the manuscript:

Plant microbiome-dependent immune enhancing action of *Echinacea purpurea* is enhanced by soil organic matter content

Author list:

Mona H. Haron, Heather L. Tyler, Suman Chandra, Rita M. Moraes, Colin R. Jackson, Nirmal D. Pugh, David S. Pasco

Supplementary Table S1. Analysis of community similarity (as assessed based on presence-absence of bacterial OTUs, Jaccard, or relative abundance of OTUs, theta) among the different cultivation conditions tested using ANOSIM. Percent organic matter in soil T1 (67.5%) – T2 (23.2%) – T3 (10.4%) – T4 (5.6%) – T5 (2.5%), soil nitrogen fertilization T1 (200 Kg/h) – T2 (150 Kg/h) – T3 (100 Kg/h) – T4 (50 Kg/h) – T5 (0 Kg/h) and soil moisture field capacity T1 (full field) – T2 (3/4 of field) – T3 (1/2 field).

**Plant part**	**Soil condition**	**p value**		**Jaccard Pairwise p value**			
		Jaccard	Theta	T1 vs T5	T1 vs T4	T2 vs T5	T2 vs T3
**Root**	Organic matter	<0.001*	0.051	0.001*	0.007	0.012	-
	Nitrogen	0.098	0.409	-	-	-	-
	Moisture content	0.477	0.333	-	-	-	-
**Aerial**	Organic matter	<0.001*	0.17	0.003*	0.029	0.007	-
	Nitrogen	0.079	0.834	-	-	-	-
	Moisture content	0.009*	0.787	-	-	-	0.009

* Statistically significant difference among treatments in each condition using an alpha of 0.05.
